# Supplementary material for: Adaptive 3D descattering with a dynamic synthesis network
Source: Light Sci Appl. 2022 Feb 24;11:42. doi: 10.1038/s41377-022-00730-x (PMC8873471; doi:10.1038/s41377-022-00730-x)
Supplement: Supplementary file 1 — Supplementary file of revision 4 [file 41377_2022_730_MOESM1_ESM.pdf]

Supplementary information for:

Adaptive 3D descattering with a dynamic synthesis network

Waleed Tahir[1,⋆], Hao Wang[1,⋆], Lei Tian[1,2,*]

[1] Department of Electrical and Computer Engineering, Boston University, Boston, MA 02215, USA.

[2] Department of Biomedical Engineering, Boston University, Boston, MA 02215, USA.

⋆ Equal contribution. * Correspondence: leitian@bu.edu, Tel.: (617) 353-1334

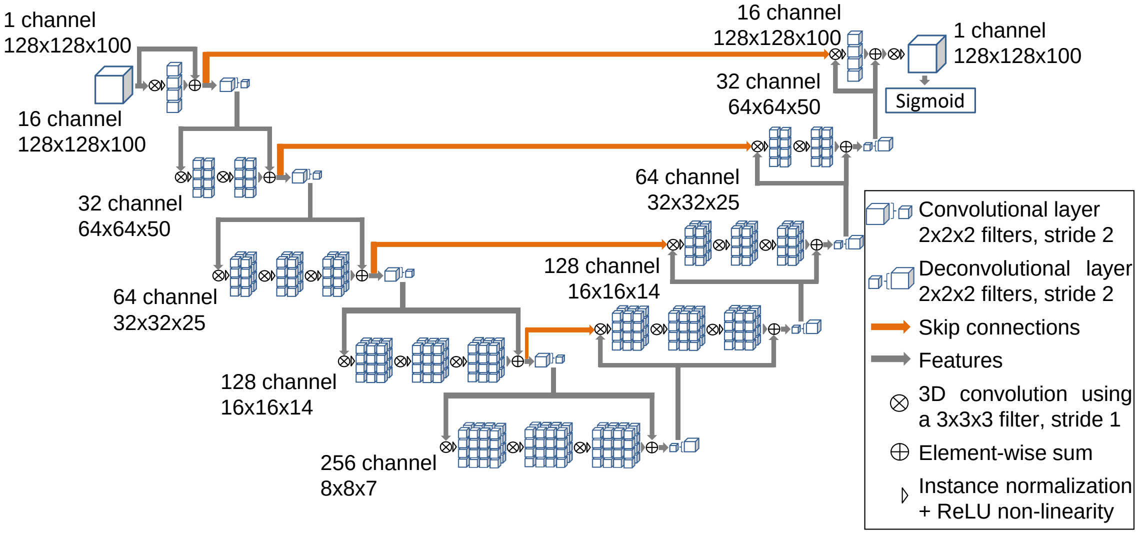

Figure S1: **Network architecture of the expert DNN.** The expert DNN builds on the V-net framework, which is a 3D DNN with an encoder-decoder framework. The encoder extracts multi-scale features using 3D convolution kernels of size $3 \times 3 \times 3$ with stride 1. Each layer in the encoder decreases the spatial dimensions by $2\times$ while doubling the number of channels. Spatial feature maps at different scales from the encoder are forwarded to the decoder via skip connections for preserving high resolution information. The bottleneck layer contains the "latent code" of size $8 \times 8 \times 7$ with 256 channels. The decoder also contains multiple 3D convolution layers with $3 \times 3 \times 3$ convolution kernels, and convolutional upsampling, with the additional incorporation of high resolution features from the skip connections. The single-channel output from the decoder is converted to a probability map using the sigmoid layer, in which each voxel value represents the likelihood of the voxel belonging to a particle (vs. belonging to the background).

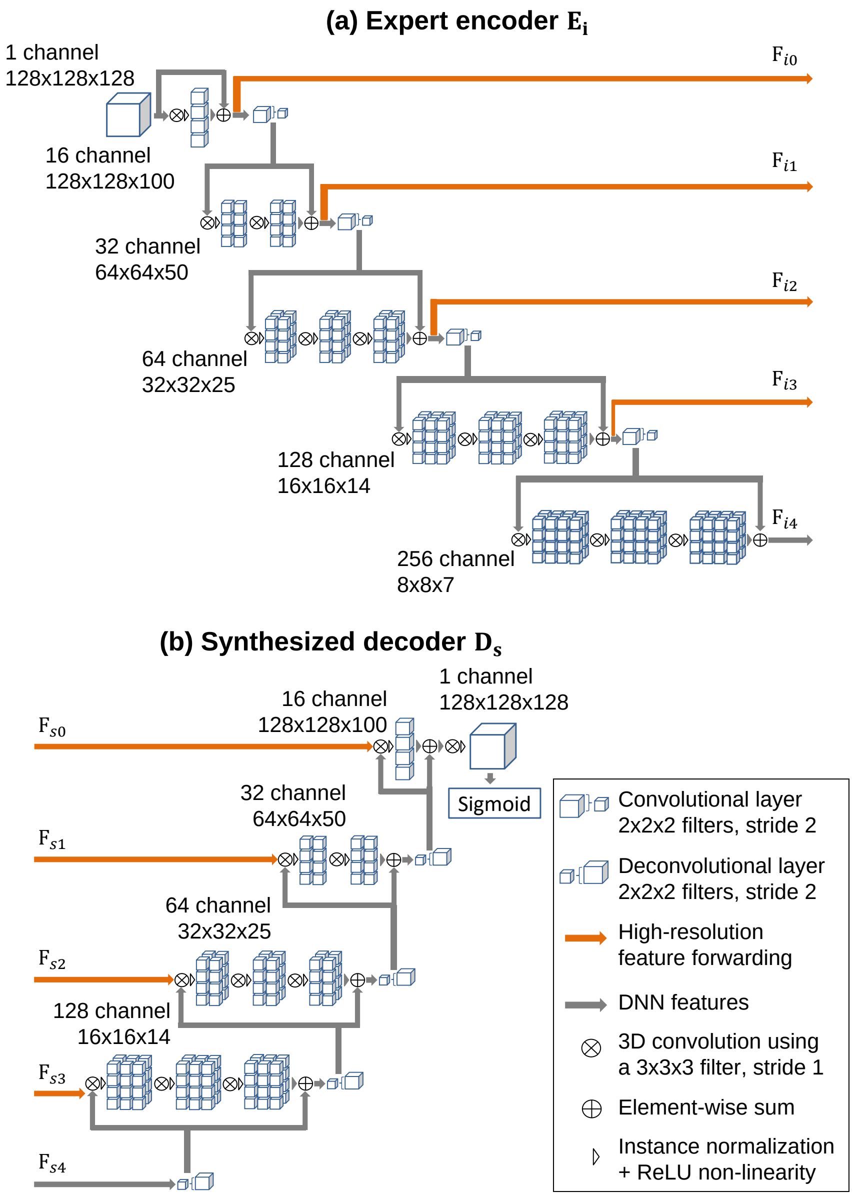

Figure S2: **Network architecture of the expert encoder** E_i **and synthesized decoder** D_s **within the DSN.** (a) The architectures of all the encoders E_i ($i \in \{1, 2, 3\}$) are identical, and are derived from a modified V-net. The encoders extract features from the 3D input, which are then used by the decoder for generating the DNN output. Since V-net uses multi-scale feature forwarding by skip connections, we record the encoder extracted features at each spatial scale (denoted by $\mathbf{F}_{i0}$–$\mathbf{F}_{i3}$), together with the latent feature map $\mathbf{F}_{i4}$. This combined set of feature maps is labeled as $\mathbf{F}_i = [\mathbf{F}_{i0}; \mathbf{F}_{i1}; \mathbf{F}_{i2}; \mathbf{F}_{i3}; \mathbf{F}_{i4}]$ associated with the corresponding encoder E_i. (b) The architecture of the synthesized decoder D_s is derived from the modified V-net decoder. Each network parameter in D_s is a weighted sum of the corresponding parameters in the three expert decoders D_i: $\mathrm{D}_s = \sum_{i=1}^{3} \alpha_i \mathrm{D}_i$. Once D_s is synthesized, it decodes the corresponding synthesized multi-scale feature maps $\mathbf{F}_s = [\mathbf{F}_{s0}; \mathbf{F}_{s1}\mathbf{F}_{s2}; \mathbf{F}_{s3}; \mathbf{F}_{s4}]$ for generating the output, where $\mathbf{F}_{sx} = \sum_{i=1}^{3} \alpha_i \mathbf{F}_{ix}$, and $x \in \{0, 1, 2, 3, 4\}$ indices the feature set.

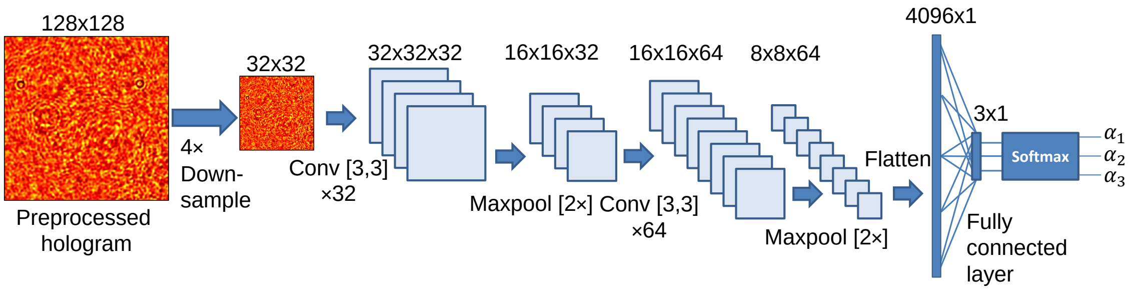

Figure S3: **Gating network structure.** The GTN follows the VGG structure to predict the synthesis weights α_i. The input hologram is first downsampled by $4\times$ by selecting every 4th pixel along each axes. Next, the GTN extracts multi-scale spatial features from the 2D hologram using two 2D convolutional layers, each containing a convolution with a bank of 3×3 kernels, followed by $2\times$ maxpooling to decrease the spatial dimensions. The first layer contains 32 channels; the second layer contains 64 channels. After the convolutional layers, the $8\times8\times64$ feature map is flattened and then passed through a fully connected layer. The output is a 3×1 vector representing the three synthesis weights $\{\alpha_1, \alpha_2, \alpha_3\}$. The sum of the weights are enforced to be unity, i.e. $\sum_{i=1}^{3} \alpha_i = 1$ through the use of the softmax nonlinear activation function at the last layer.

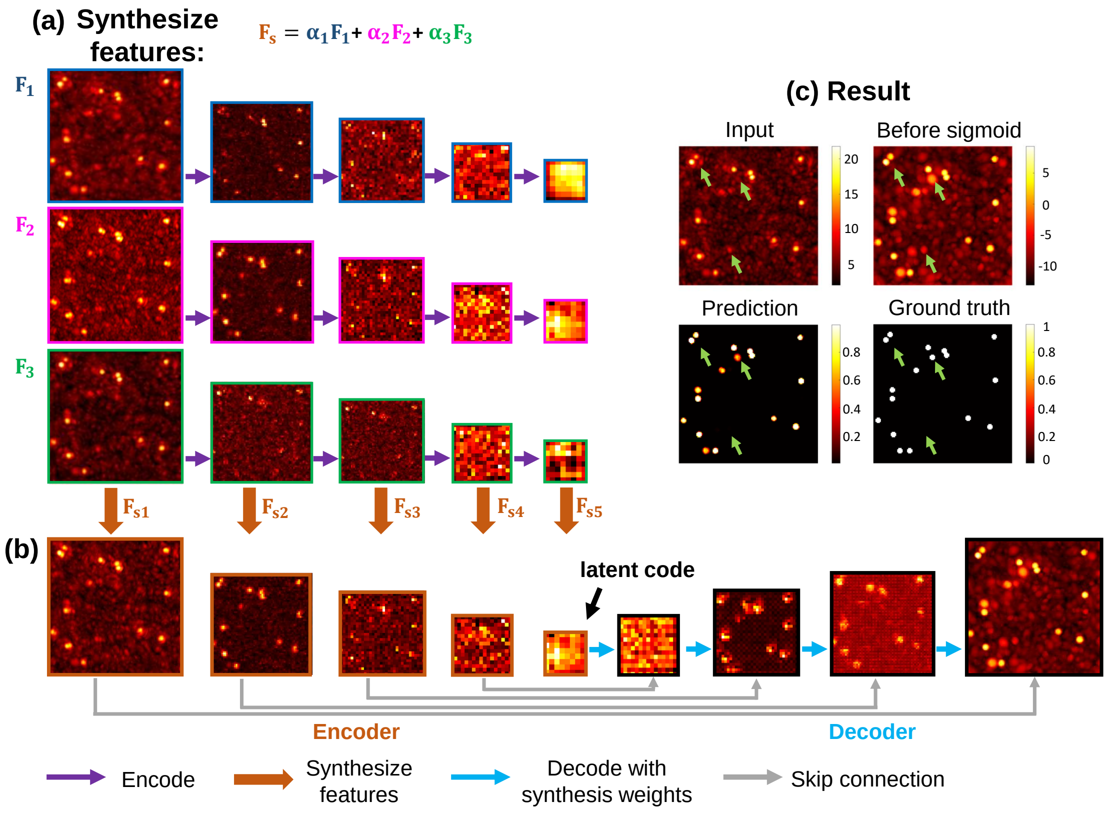

Figure S4: **Schematic diagram of the feature map flow in the DSN.** We show the maximum z-projection of an example 3D feature map. (a) The feature maps extracted by the three expert encoders. (b) The feature maps in the synthesized DSN. The synthesized encoder features are directly concatenated to the decoder by the skip connections. (c) The input and output of the DSN.

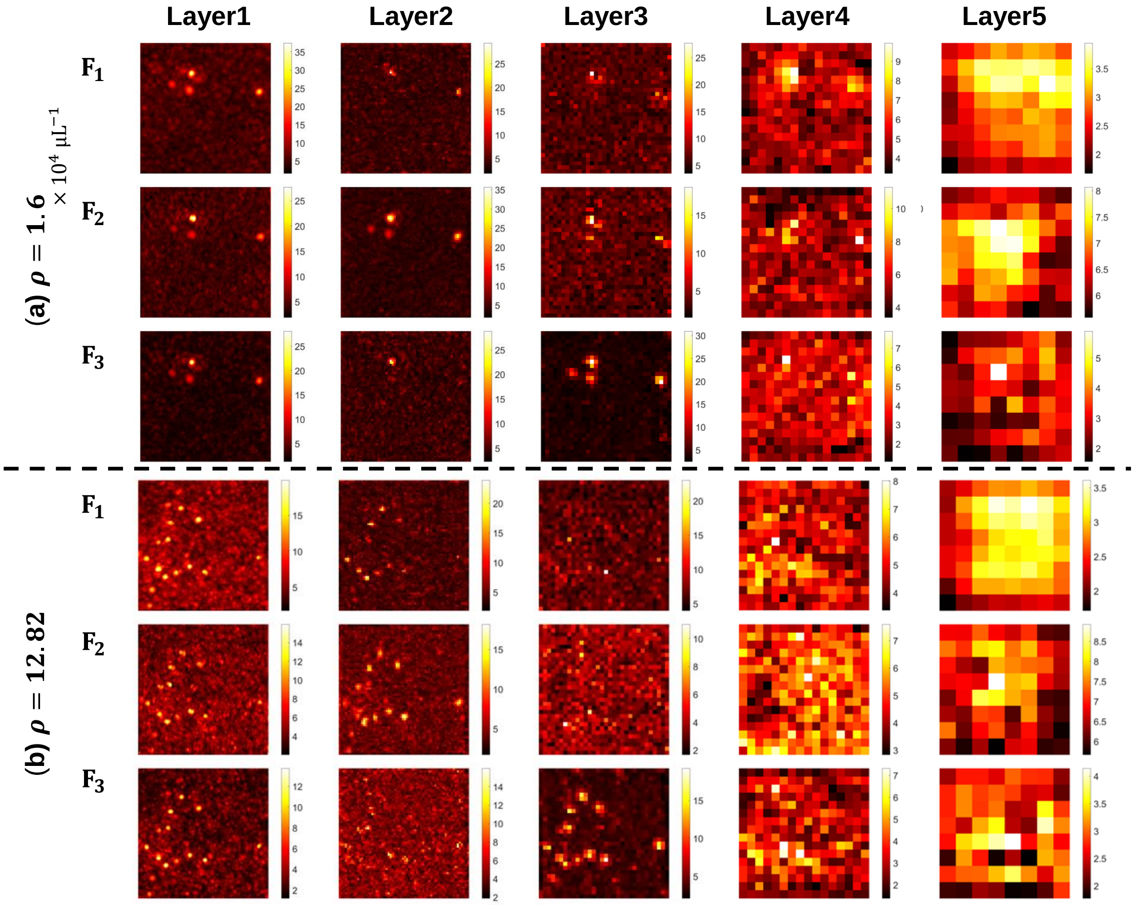

Figure S5: **DSN Feature maps at different particle densities.** The feature maps (shown in z-projects) of a low density and a high density are shown. $\mathbf{F}_1, \mathbf{F}_2, \mathbf{F}_3$ represent the three expert encoders within the DSN. Layer1–Layer5 represent the encoder layers within the DSN, the same as Fig. S4(a).

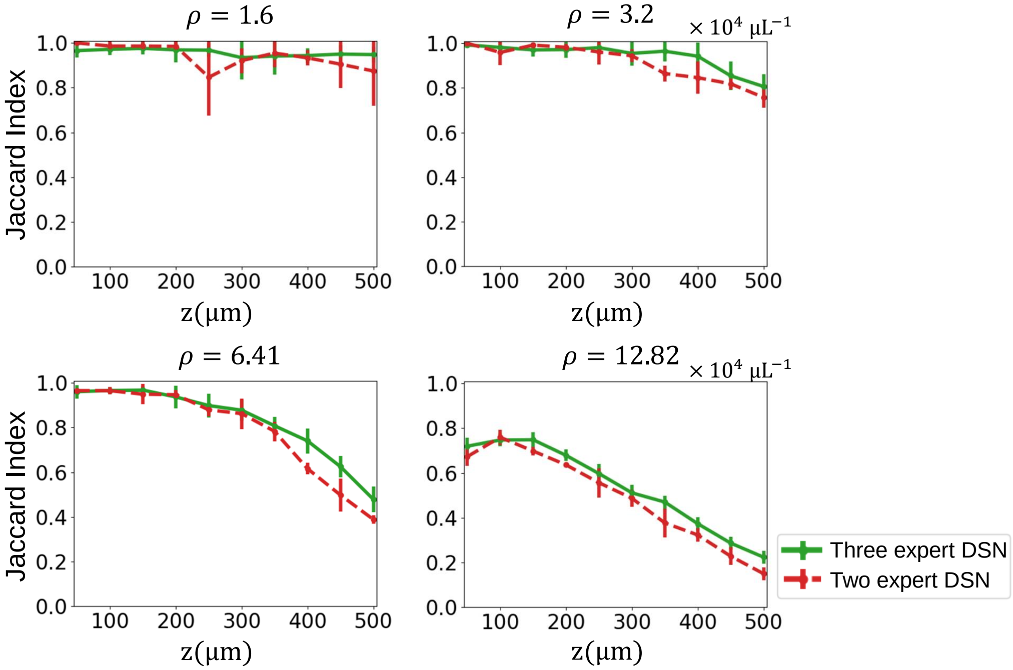

Figure S6: **Performance comparison: two vs three expert encoder-decoder pairs within the DSN.** We compare the performance of the DSN with two (in dashed red) and three (in solid green) experts on the test set which has the same particle size, refractive index contrast and density as the training set. It is evident that the three-expert DSN performs better especially for higher particle densities ($\rho \geq 3.2 \times 10^4$ particles μL^{-1}). The results highlight the significance of the extra degrees of freedom provided by the additional expert within the DSN.

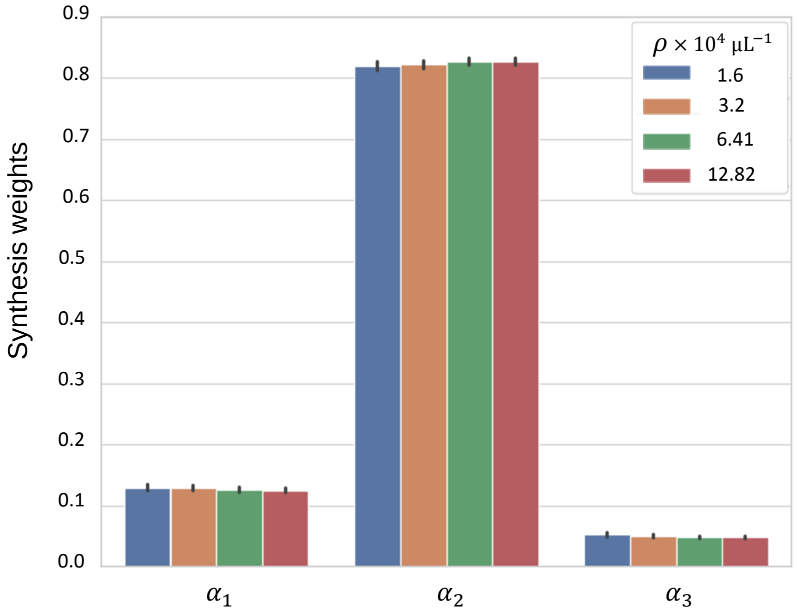

Figure S7: **The synthesis weights with Xavier-random initialization of the experts.** The synthesis weights of four different scattering densities (ρ) predicted by the GTN, co-trained with the random initialized experts, are shown for the simulated testing set (refractive index is 0.26, particle diameter is 1.0 μm). Different expert DNNs have different weights α, which suggest that they contribute differently to the descattering. The larger values of α_2 indicate the major contributions of $\mathbf{F}_2$ and D_2 to the DSN. The small values of α_3 indicates the fine-tuned contributions from $\mathbf{F}_3$ and D_3 to the DSN. The synthesis weights are consistent for a given density and tailored to each input, as quantified by the mean and standard deviation for each case. The general trend of the synthesis weights are similar to those obtained from the DSN using the pre-trained weight initialization scheme, shown in the Figure 6. As the particle density increases, α_1, α_3 decreases while α_2 increases, which indicates that E_2 are important for the DSN to adapt to higher density cases. The differences in the weight values compared with Figure 6 are expected from the different initialization schemes, the stochastic training process, and the severe ill-posedness of problem.

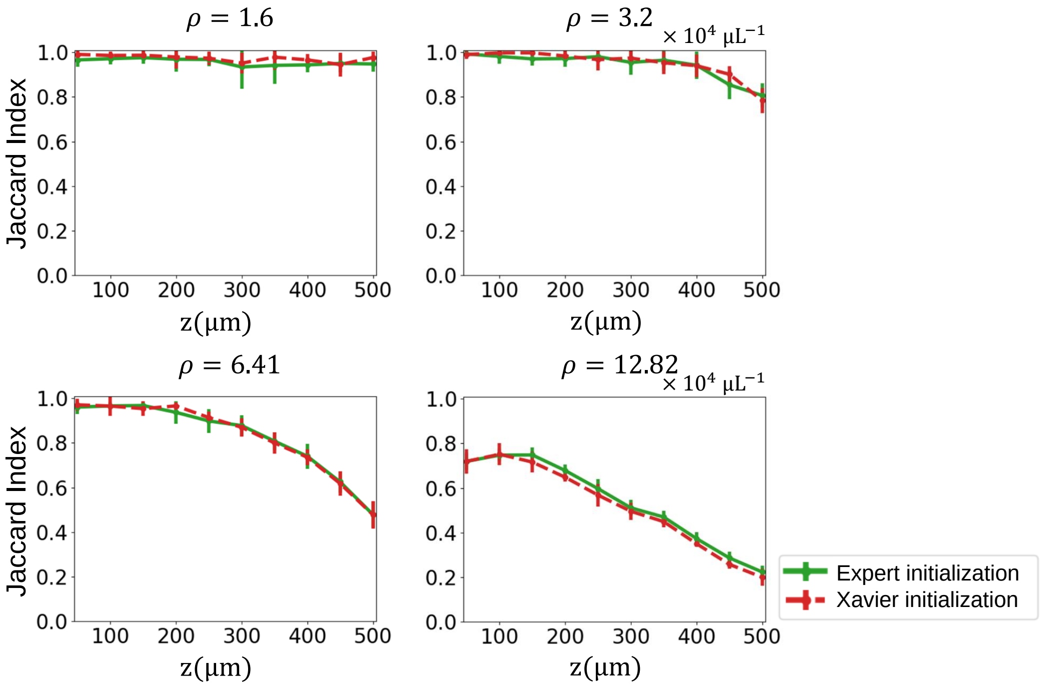

Figure S8: **Performance comparison: expert vs Xavier-random initialization of the encoders and decoders within the DSN.** Particle localization performance is quantitatively compared between the DSNs that are trained using two initialization schemes, including the pre-trained expert weights (solid green) and Xavier random weights (dashed red) using the Jaccard Index (JI). Each subplot indicates the results on the test set (refractive index is 0.26, particle diameter is 1.0 μm) at the particle density labeled above each plot. The two initialization schemes provide almost the same performance.

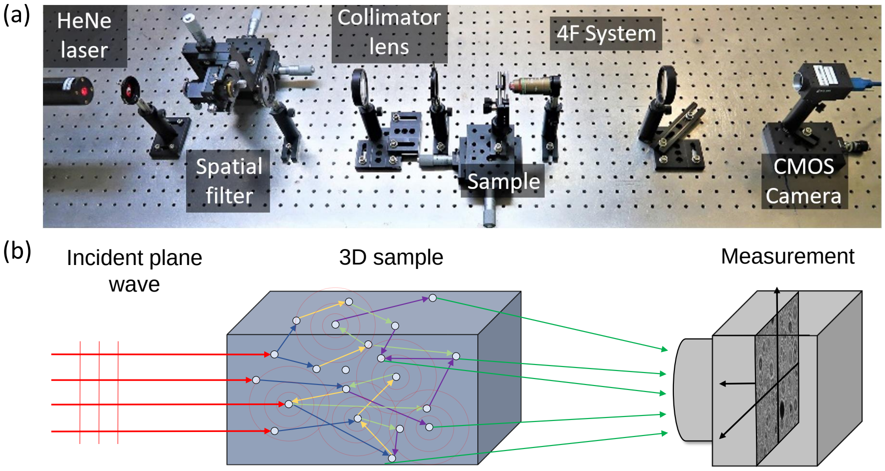

Figure S9: **Experimental setup and schematics.** (a) An inline holography setup consists of a collimated HeNe laser (632.8 nm, 500:1 polarization ratio, Thorlabs HNL210L) for illumination and a 4F system consisting of a 20× objective lens (0.4 NA, CFI Plan Achro) and a 200 mm tube lens for imaging. A CMOS sensor (FLIR GS3-U3-123S6M-C) is used to record the holograms. The 3D sample consists of polystyrene microspheres with diameter 0.994±0.021 μm (Thermofisher Scientific 4009A) suspended in water held in a quartz-cuvette with inner dimensions 40 mm × 40 mm × 0.5 mm. (b) A plane-wave is incident on the 3D sample containing distributed particles. The field undergoes multiple scattering and then propagates to the hologram plane.

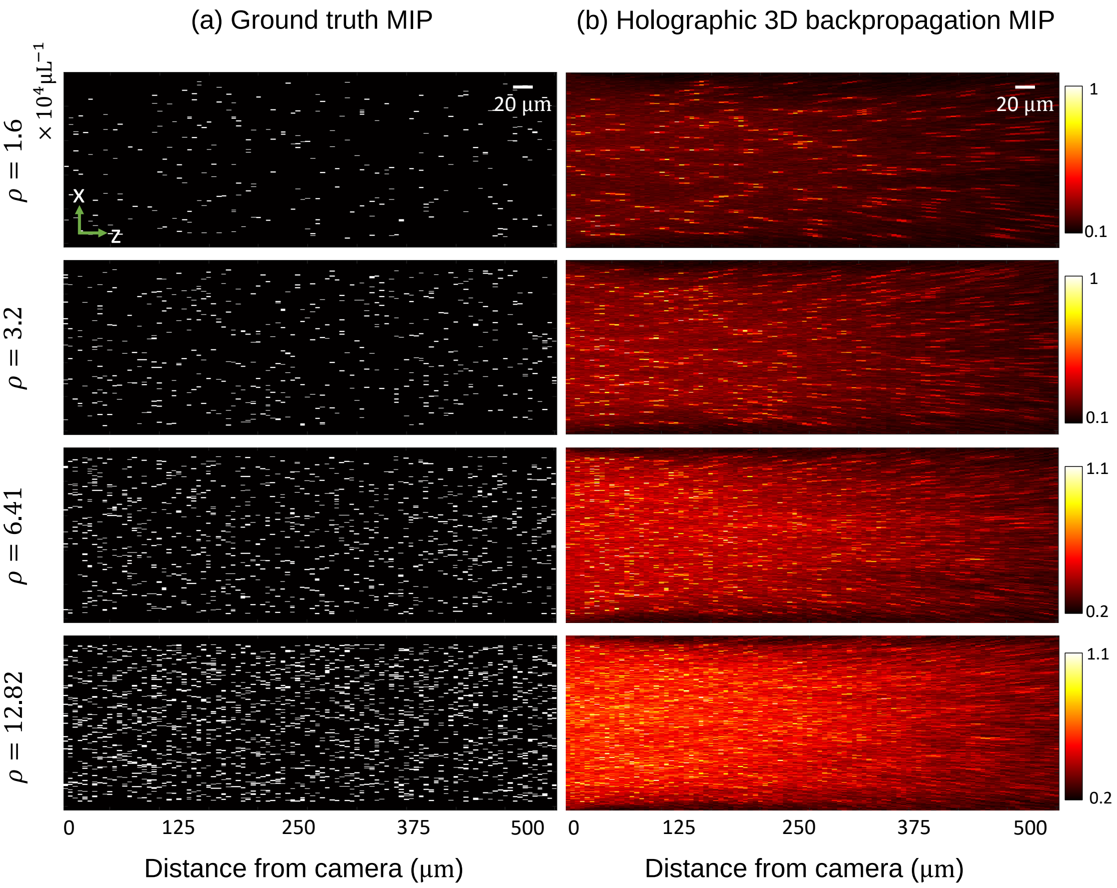

Figure S10: **Scatterer-density and depth-dependent artifacts in representative holographic 3D backpropagation volumes used in the DSN training.** Maximum intensity $y-$projections of (a) ground-truth volume (particles shown in white, background in black), and (b) holographically backpropagated volume. Characteristic scatterer-density and depth-dependent artifacts are clearly visible. As the particle density increases, more severe scattering artifacts throughout the volume are shown. More elongation and reduced intensity in the backpropagated particle traces are observed at deeper depths due to reduced effective light collection angular range.

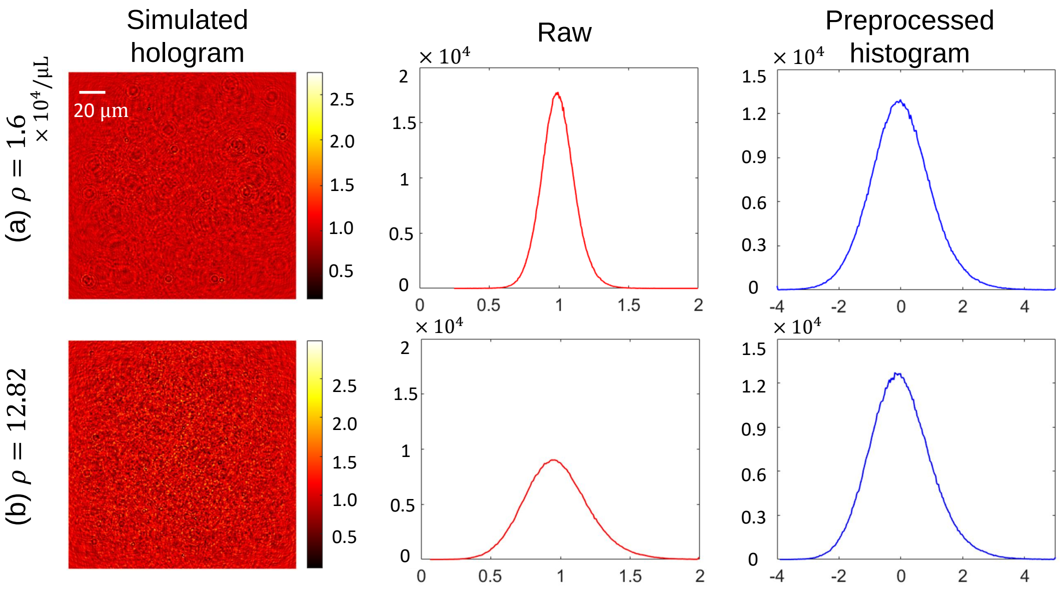

Figure S11: **The preprocessing result of holograms.** (a) A simulated hologram at a low density ($\rho = 1.6 \times 10^4$ particles μL^{-1}), refractive index contrast 0.26, particle size 1 μm), the raw intensity histogram, and the histogram after preprocessing. (b) A simulated holograms at a high density ($\rho = 12.82 \times 10^4$ particles μL^{-1}) particles, refractive index contrast 0.26, particle size 1 μm), the raw intensity histogram, and the histogram after preprocessing. The raw histograms from the two particle densities are obviously different in both mean and standard deviation. After preprocessing, both histograms approximately follow the same Gaussian distribution.

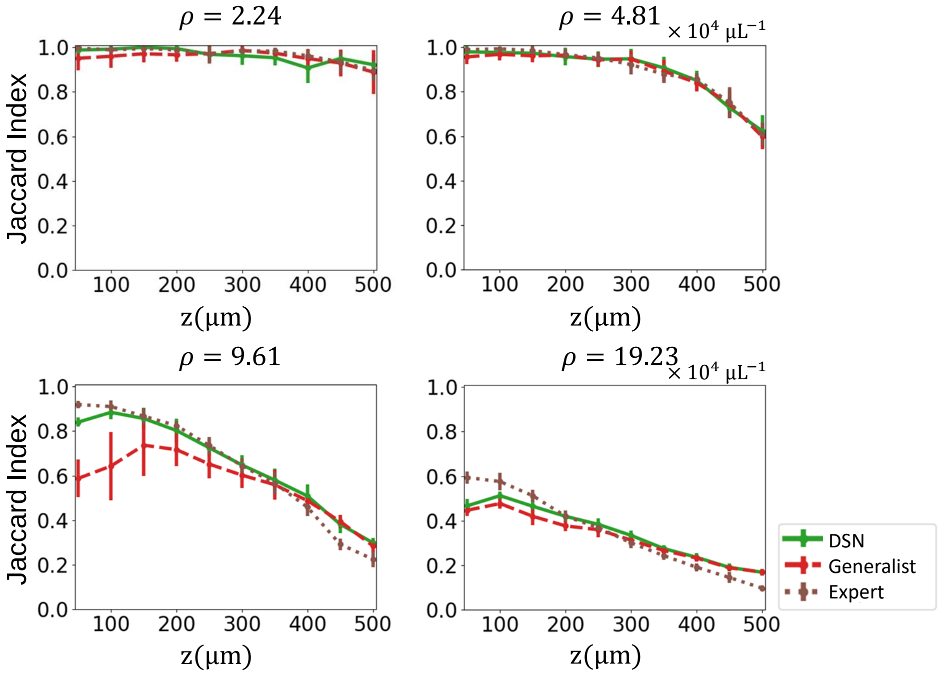

Figure S12: **Comparison between the DSN and the baseline generalist on unseen densities.** Each subplot indicates the results on the testing data at the particle density labeled above each plot. 'Expert ρ' represents the expert DNN trained on the data with a particle density ρ ($\times 10^4$ particles μL^{-1}). The DSN and the baseline generalist are trained using the same data from four other densities, as detailed in the main text. The DSN provides markedly higher accuracy than the generalist, in particular for high particle densities ($\rho \geq 9.61 \times 10^4$ particles μL^{-1}).

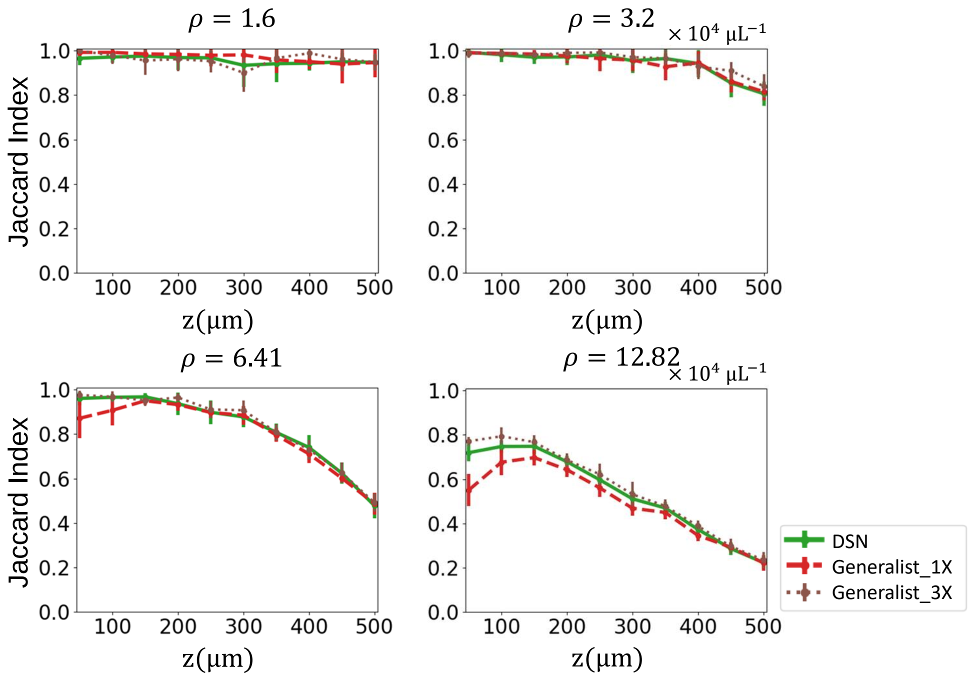

Figure S13: **Comparison between the DSN and the generalist networks on seen scattering densities.** Each subplot indicates the results on the testing data at the particle density labeled above each plot with refractive index contrast 0.26 and particle size 1 μm. The DSN, the baseline generalist (labeled as Generalist_1×) and the 3× generalist (labeled as Generalist_3×) are trained using the same data from four other densities, as detailed in the main text. The DSN and the 3× generalist perform similarly in the three lower densities. For the highest density, the 3× generalist performs slightly better than the DSN at the shallow depths.

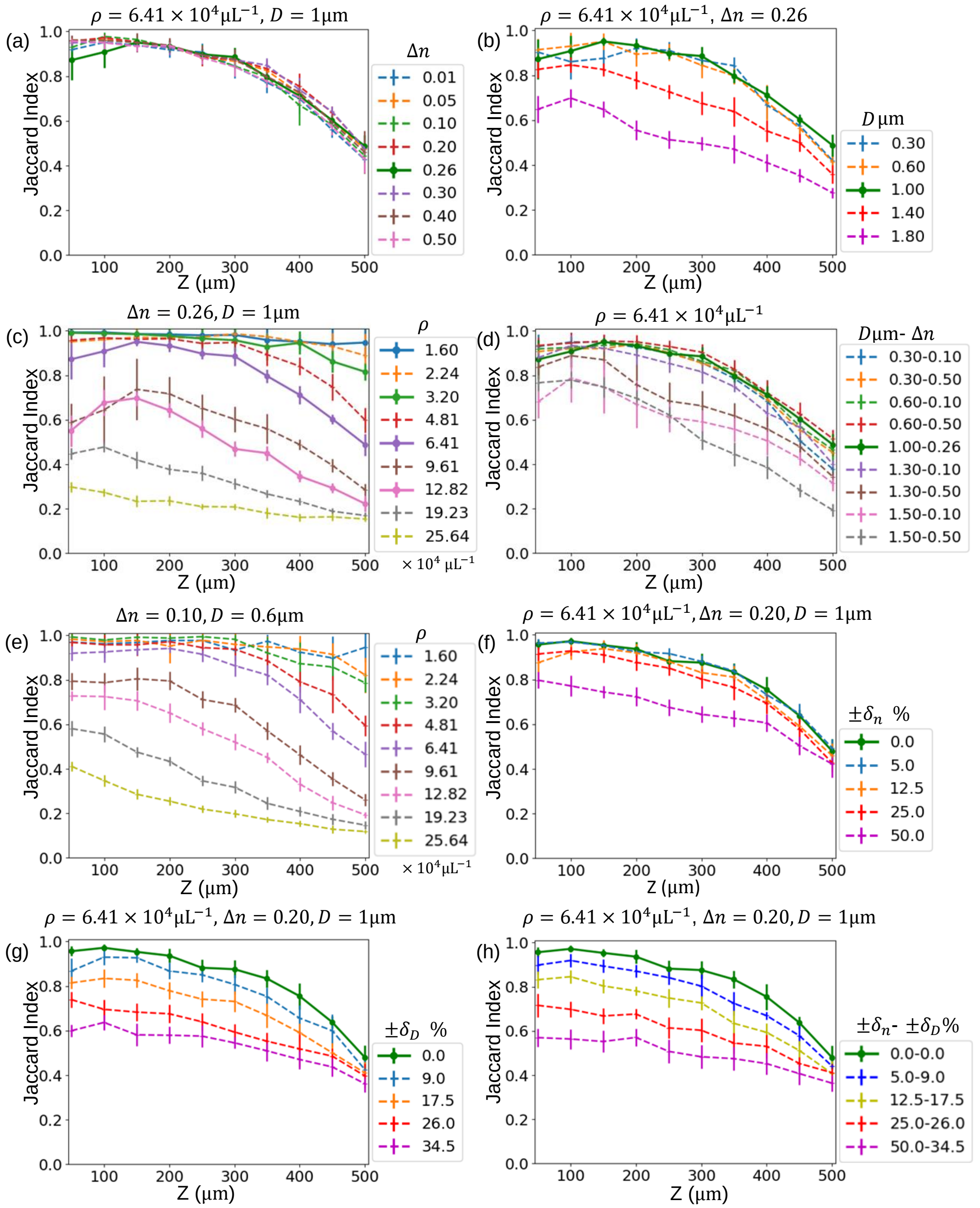

Figure S14: **Generalization of the baseline generalist to unseen scattering conditions.** The baseline seen cases are shown in solid lines; the "unseen" test conditions are in dashed lines. The testing cases are identical to that for the DSN in Fig. 4, including: (a) unseen refractive index contrast; (b) unseen particle size; (c) unseen particle density; (d) unseen refractive index contrast and particle size; (e) unseen refractive index contrast, particle size and density; (f) Uniformly distributed random refractive index contrast, δ_n% denotes the variation range with respect to the central refractive index contrast; (g) Uniformly distributed random particle size, δ_d% denotes the variation range with respect to the central size; (h) Uniformly distributed random refractive index and particle size. In (f)-(h), the green dash-dotted line is the baseline unseen case at $\rho = 6.41 \times 10^4$ particles μL^{-1}, with a fixed refractive index contrast $\Delta n = 0.20$ and a fixed particle size $D = 1.0$ μm.

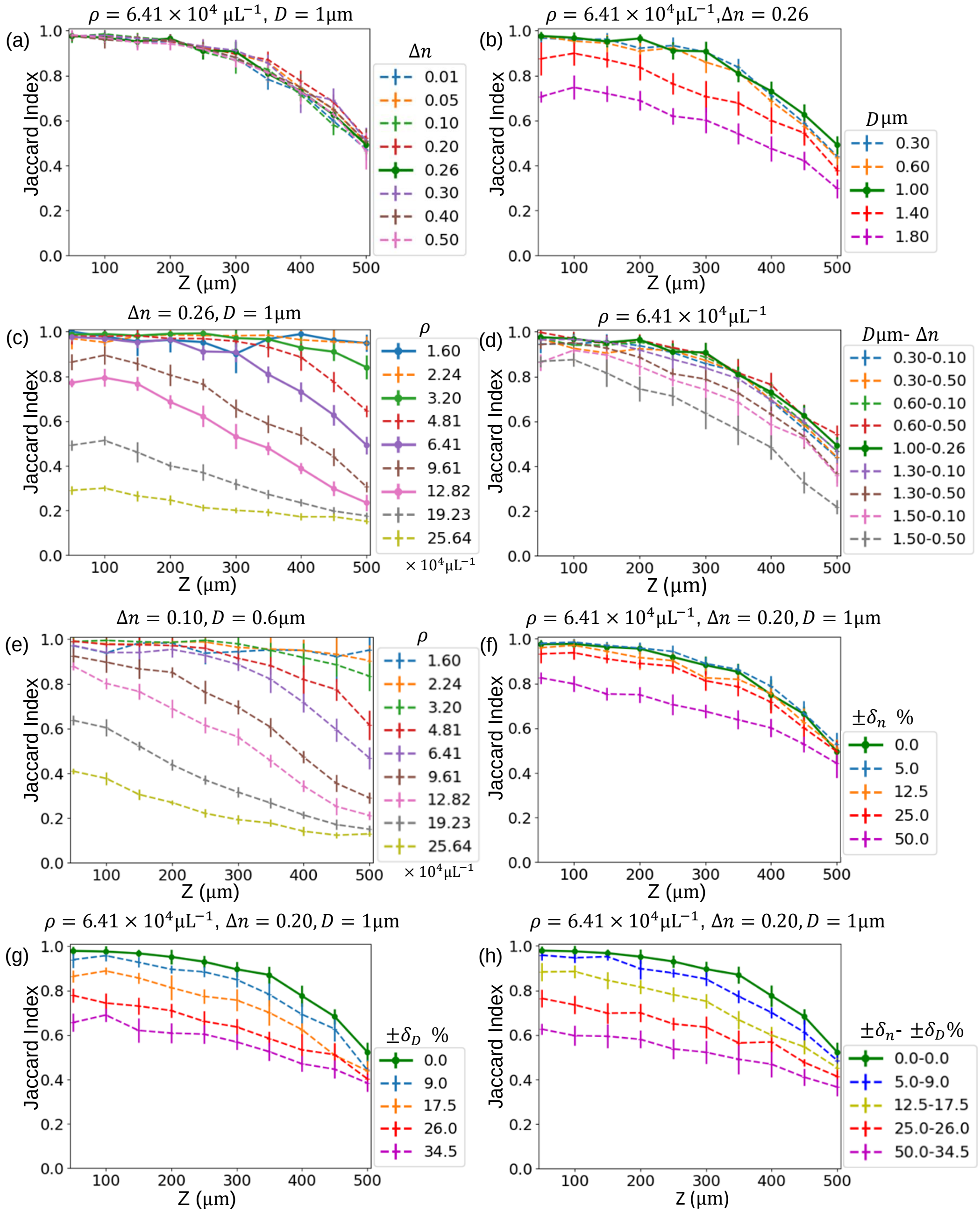

Figure S15: **Generalization of the 3× generalist to unseen scattering conditions.** The baseline seen cases are shown in solid lines; the "unseen" test conditions are in dashed lines. The testing cases are identical to that for the DSN in Fig. 4, including: (a) unseen refractive index contrast; (b) unseen particle size; (c) unseen particle density; (d) unseen refractive index contrast and particle size; (e) unseen refractive index contrast, particle size and density; (f) Uniformly distributed random refractive index contrast, $\delta_n\%$ denotes the variation range with respect to the central refractive index contrast; (g) Uniformly distributed random particle size, $\delta_d\%$ denotes the variation range with respect to the central size; (h) Uniformly distributed random refractive index and particle size. In (f)-(h), the green dash-dotted line is the baseline unseen case at $\rho = 6.41 \times 10^4$ particles μL^{-1}, with a fixed refractive index contrast $\Delta n = 0.20$ and a fixed particle size $D = 1.0$ μm.

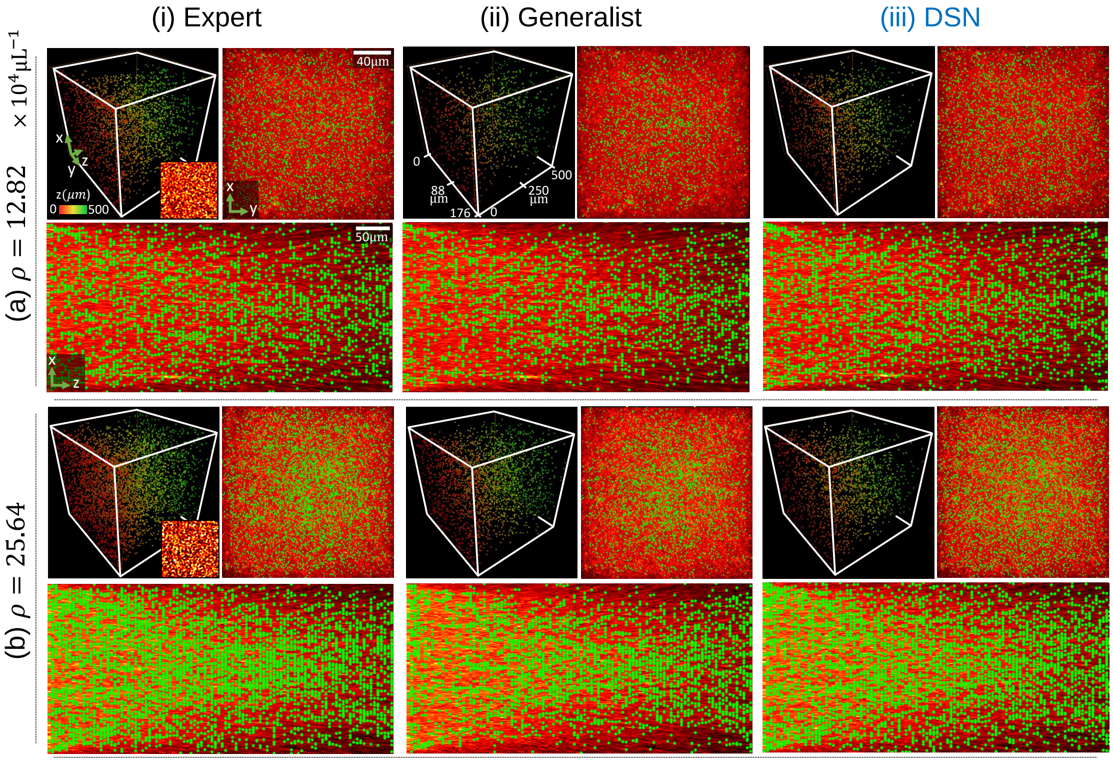

Figure S16: **Experimental results from the simulator-trained DNNs.** Particle 3D localization is shown for experimentally measured holograms using the simulator-trained (i) expert, (ii) generalist, and (iii) DSN networks at two higher particle densities. Each panel shows (Top left) the 3D rendering of the localization result with depth color-coded particles, with an inset showing a zoom-in of the measured hologram, (Top right) the maximum intensity $z-$ projection, and (Bottom) the $y-$projection of the DSN's 3D localization result (in green), overlaid on the respective $y-$ and $z-$projections of the corresponding holographic backpropagated volumes.

Table S1: **The numbers of True Positive (TP), False Positive (FP), False Negative (FN)** (mean $\pm$ standard deviation) **for each particle number.**

		**Expert**	
**Particle number**	**TP**	**FP**	**FN**
**250**	247.2 ± 2.9	3.0 ± 1.3	2.8 ± 2.9
**500**	477.6 ± 4.6	3.9 ± 3.2	22.4 ± 4.6
**1000**	863.4 ± 14.4	93.9 ± 15.3	136.6 ± 14.4
**2000**	1239.4 ± 23.3	518.0 ± 46.3	760.6 ± 23.3
		**Generalist**	
**Particle number**	**TP**	**FP**	**FN**
**250**	248.5 ± 2.3	7.8 ± 5.9	1.5 ± 2.3
**500**	482.4 ± 10.0	21.3 ± 8.2	17.6 ± 10.0
**1000**	864.3 ± 16.8	140.3 ± 13.3	135.7 ± 16.8
**2000**	1215.4 ± 24.6	630.4 ± 27.3	784.6 ± 24.6
		**DSN**	
**Particle number**	**TP**	**FP**	**FN**
**250**	244.8 ± 3.6	13.7 ± 6.0	5.2 ± 3.6
**500**	479.4 ± 6.2	15.7 ± 7.4	20.6 ± 6.2
**1000**	868.2 ± 4.8	65.7 ± 11.5	131.8 ± 4.8
**2000**	1246.3 ± 23.2	436.3 ± 29.7	753.7 ± 23.2

Table S2: **The kernel size and number of input, ouput channels of the Generalist network.**

				**Expert/Generalist**		
**Layer number**	**x**	**y**	**z**	**Input channel**	**Output channel**	**Total parameter**
1	3	3	3	1	16	448
2	3	3	3	16	16	7168
3	2	2	2	16	32	4608
4	3	3	3	32	32	28672
5	2	2	2	32	64	18432
6	3	3	3	64	64	114688
7	2	2	2	64	128	73728
8	3	3	3	128	128	458752
9	2	2	2	128	256	294912
10	3	3	3	256	256	1835008
11	2	2	2	128	256	294912
12	3	3	3	128	128	458752
13	2	2	2	64	128	73728
14	3	3	3	64	64	114688
15	2	2	2	32	64	18432
16	3	3	3	32	32	28672
17	2	2	2	16	32	4608
18	3	3	3	16	16	7168
19	1	1	1	16	1	32
**Total**				**1233**	**1713**	**3837408**

Table S3: **The kernel size and number of input, ouput channels of the 3× Generalist.**

				3X Generalist		
**Layer number**	**x**	**y**	**z**	**Input channel**	**Output channel**	**Total parameter**
1	3	3	3	1	28	784
2	3	3	3	28	28	21952
3	2	2	2	28	56	14112
4	3	3	3	56	56	87808
5	2	2	2	56	112	56448
6	3	3	3	112	112	351232
7	2	2	2	112	224	225792
8	3	3	3	224	224	1404928
9	2	2	2	224	448	903168
10	3	3	3	448	448	5619712
11	2	2	2	224	448	903168
12	3	3	3	224	224	1404928
13	2	2	2	112	224	225792
14	3	3	3	112	112	351232
15	2	2	2	56	112	56448
16	3	3	3	56	56	87808
17	2	2	2	28	56	14112
18	3	3	3	28	28	21952
19	1	1	1	16	1	32
	**Total**			**2145**	**2997**	**11751408**
